# Eradication of breast cancer with bone metastasis by autologous formalin-fixed tumor vaccine (AFTV) combined with palliative radiation therapy and adjuvant chemotherapy: a case report

**DOI:** 10.1186/1477-7819-11-127

**Published:** 2013-06-04

**Authors:** Fumito Kuranishi, Tadao Ohno

**Affiliations:** 1 Department of Surgery, Innoshima-Ishikai Hospital, Innoshima, Onomichi, Hiroshima, 722-2211, Japan; 2 Cell-Medicine, Inc., 2-1-6 Sengen, Tsukuba, Ibaraki, 305-0074, Japan; 3 Advanced Research Institute for Science and Engineering, Waseda University, Shinjuku-ku, Tokyo, 169-8555, Japan

**Keywords:** Tumor vaccine, Mammary carcinoma, Bone metastasis, Eradication

## Abstract

Skeletal metastasis of breast carcinoma is refractory to intensive chemo-radiation therapy and therefore is assumed impossible to cure. Here, we report an advanced case of breast cancer with vertebra-Th7 metastasis that showed complete response to combined treatments with formalin-fixed autologous tumor vaccine (AFTV), palliative radiation therapy with 36 Gy, and adjuvant chemotherapy with standardized CEF (cyclophosphamide, epirubicin, and 5FU), zoledronic acid, and aromatase inhibitors following mastectomy for the breast tumor. The patient has been disease-free for more than 4 years after the mammary surgery and remains well with no evidence of metastasis or local recurrence. Thus, a combination of AFTV, palliative radiation therapy, and adjuvant chemotherapy may be an effective treatment for this devastating disease.

## Background

Severe pain from secondary conditions encumbers the mobility of cancer patients and therefore compromises their quality of life. Skeletal metastasis is one such condition, which generally complicates the treatment of cancers. Metastasized breast carcinomas are frequently found in the rib, vertebrae, or pelvis, and are generally refractory to radiation therapy combined with standardized chemotherapy comprising cyclophosphamide, epirubicin, and 5FU (CEF) [1]. Zoledronic acid and aromatase inhibitors are not expected to cure the breast carcinomas with bone metastasis, although they are effective in restraining further progression of the cancer and can significantly improve overall survival [2] and disease-free survival [3], respectively. However, we have observed during a period of 10 years until the end of 2006 more than 300 cases of mammary carcinoma with bone metastasis, and all patients showed downhill course resulting in fatality despite administration of intensive chemo-radiation therapy. Here, we report a case of breast carcinoma with apparent bone metastasis that showed a complete response to combined treatments with formalin-fixed autologous tumor vaccine (AFTV), palliative radiation therapy, and adjuvant chemotherapy.

## Case presentation

**Figure 1:**
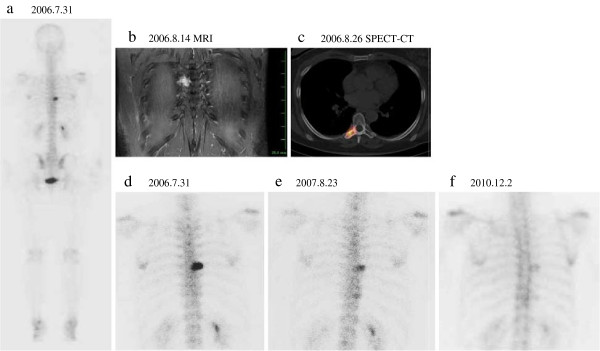
**Images before and after autologous formalin-fixed tumor vaccine (AFTV) treatment, palliative radiotherapy, and adjuvant chemotherapy. a**) ^99m^Tc-whole body scan, **b**) magnetic resonance image (MRI), and **c**) single photon emission computed tomography (SPECT-CT) image before surgical resection of the primary tumor on 31 June 2006. **d**) Expanded image of ‘a’ at the site of vertebra Th7 on 31 June 2006, **e**) 1 year later, and **f**) 4 years later at the same site.

A 52-year old woman presented with a right breast lump (4.2 × 3.0 × 4.5 cm), which was diagnosed as breast carcinoma by mammography, ultrasonography, ultrasonography-guided aspiration biopsy cytology (ABC), computed tomography (CT), and magnetic resonance imaging (MRI). Further, by bone scintigraphy with ^99m^Tc, we also found a strong uptake at the location of vertebra Th7 (Figure 1a,d), which was the cause of back pain and corresponded to a metastasized tumor, 3.0 cm in diameter, as assessed by MRI and CT (Figure 1b,c). We performed right modified radical mastectomy on 7 August 2006, and pathologically identified the tumor as mucinous carcinoma. None of the 16 tumor-draining lymph nodes tested so far revealed any carcinoma cells.

Since the patient strongly desired to be treated with a new tumor vaccine, we prepared AFTV with 3.0 g of formalin-fixed and paraffin-embedded breast carcinoma tissues as previously described [4]. The vaccination started on 27 August 2006, and continued once a week for 3 weeks. Concomitantly, palliative radiation therapy was carried out from 28 August to 12 September. A total dose of 36 Gy of X-ray (3 Gy per day, 12 fractions) was given to the local site of bone metastasis. The treatment course was uneventful; there were no adverse effects.

Delayed-type hypersensitivity (DTH) response [4], tested with the patient’s fixed tumor fragments but without any immunoadjuvant, was negative (no erythema was observed) before the first AFTV injection. However, after leaving our hospital on 15 September 2006, the DTH response became pseudo-positive with presence of a 5 × 5 mm erythema. The DTH test was re-performed on 14 February 2012 (5.4 years later) and was once again pseudo-positive with presence of a 7 × 7 mm erythema.

Starting from 24 October 2006, the patient was also given adjuvant chemotherapy comprising six courses of CEF, zoledronic acid, and aromatase inhibitors (Table 1). The high activity in vertebra Th7 shown by scintigraphy diminished a year later (Figure 1e), becoming fainter and fainter by December 2009, and stabilized to a scar-like background signal level 4 years later (Figure 1f). At present, more than 6 years after the primary surgery, the patient remains well with no evidence of metastasis or local recurrence.

**Table 1:** Details of the adjuvant chemotherapy administered to the patient following the concomitant autologous formalin-fixed tumor vaccine (AFTV) and palliative radiation treatments

**Date**	**Courses**
2006.10.24 to 2006.11.20, 2007.04.19 to 2011.11.30	Zoledronic acid, 4 mg, once a month
2006.10.31 to 2007.4.10	CEF (cyclophosphamide 100 mg/day × 14 days + once a week with 50 mg epirubicin and 500 mg 5FU, 2 weeks), 6 courses
2007.05.01 to 2008.03.15	Anastrozole, 1 mg/day
2008.03.11 to 2011.11.30	Exemestane, 25 mg/day

## Conclusions

Before encountering the present case, none of our patients had ever undergone complete eradication of breast carcinoma with bone metastasis after radiation therapy and adjuvant chemotherapy. It is well known that irradiation of bone metastases is a palliative treatment, which was concluded from results based on 16 randomized trials, 20 prospective studies, 5 retrospective studies and 22 other articles, involving a total of 8,051 patients [5]. Therefore, it is extremely rare to observe complete eradication of skeletal metastases. Particularly in the trunk area, conventional full dose irradiation (50 to 60 Gy), which may cause late radiation injury of major organs, should be avoided, and lower doses, such as 36 Gy used for pain suppression during palliative therapy, are unable to eradicate the skeletal metastases. Moreover, no adjuvant chemotherapeutic regimen has been found to effectively treat skeletal metastasis of breast carcinomas.

We have observed that X-ray irradiation upregulated glioma cell immunogenicity [6]. This phenomenon suggests that the combination of irradiation and immunotherapy is a good candidate as a therapeutic approach against malignant cells [7-9], and moreover, it has been shown that this combination treatment can enhance anti-tumor effects [10-12]. Thus, the treatment course of the present patient with the additional immunotherapy, AFTV, is considered to be well justified.

There is currently no clinically established system available for estimation of anti-tumor cellular immune reactivity. Since DTH testing is commonly used to measure specific anti-tumor cellular immune reactivity, we used it to evaluate the anti-tumor cellular immune status just before and later after AFTV treatment. The reactivity was revealed to be pseudo-positive 2 weeks after the last AFTV injection in the present case, which lasted for more than 5 years. Therefore, we assumed that a cellular immune response against the carcinoma was induced by the AFTV treatment, though weakly and very slowly, and cooperated with X-ray irradiation and chemotherapy to contribute to the eradication of skeletal metastasis of the breast carcinoma.

Breast cancer is generally not considered as just one disease, but a heterogeneous collection of distinct entities that are molecularly quite different [13]. We therefore assume that whole autologous tumor tissue should contain the full set of tumor-associated antigenic peptides derived from the heterogeneous collection within the primary tumor. In the present case, we used a mixture of both paraffin-embedded and formalin-fixed autologous tumor tissues as the source of tumor antigens.

We aimed to incorporate the original tumor antigens in the breast carcinoma as well as any possible alterations of the expressed tumor antigens in the progressive tumor cells. As a result, this approach appeared to be successful. In the context of metastatic bone disease, the principles are the same in any type of cancer, with an increasingly targeted approach to and individualization of therapy [13].

In conclusion, the outcome of the present case suggests that AFTV in combination with concomitant administration of palliative X-ray irradiation, adjuvant CEF, zoledronic acid, and aromatase inhibitors eradicates skeletal metastasis of breast carcinoma with enhancement of a specific anti-tumor immune reactivity. A larger scale clinical study is necessary to confirm the efficacy of AFTV treatment in skeletal metastases of breast carcinomas.

## Consent

Written informed consent in English and Japanese was obtained from the patient for publication of this case report and any accompanying images.

## Abbreviations

ABC: Aspiration biopsy cytology; AFTV: Autologous formalin-fixed tumor vaccine; CEF: Cyclophosphamide, epirubicin and 5FU; CT: Computed tomography; DTH: Delayed-type hypersensitivity; MRI: Magnetic resonance imaging; SPECT-CT: Single photon emission computed tomography; 5FU: 5-fluorouracil.

## Competing interests

The authors declare that they have no competing interests.

## Authors’ contributions

FK, as the principal doctor, operated on the patient, carried out the AFTV therapy, and wrote the report. TO suggested to treat the patient with AFTV and produced the vaccine and amended the report. All authors read and approved the final manuscript.

## Authors’ information

FK is affiliated with Innoshima-Ishikai Hospital. TO is the president and CEO of Cell-Medicine, Inc., a company for research and development of immunotherapy, and is a visiting professor of Waseda University.
